# Efficient Design for Integrated Photonic Waveguides with Agile Dispersion

**DOI:** 10.3390/s21196651

**Published:** 2021-10-07

**Authors:** Zhaonian Wang, Jiangbing Du, Weihong Shen, Jiacheng Liu, Zuyuan He

**Affiliations:** State Key Laboratory of Advanced Optical Communication Systems and Networks, Shanghai Jiao Tong University, Shanghai 200240, China; ee_wzn@sjtu.edu.cn (Z.W.); shenweihong@sjtu.edu.cn (W.S.); jiacheng.liu@sjtu.edu.cn (J.L.); zuyuanhe@sjtu.edu.cn (Z.H.)

**Keywords:** dispersion engineering, slot waveguide, inverse design, deep neural network, optical frequency comb

## Abstract

Chromatic dispersion engineering of photonic waveguide is of great importance for Photonic Integrated Circuit in broad applications, including on-chip CD compensation, supercontinuum generation, Kerr-comb generation, micro resonator and mode-locked laser. Linear propagation behavior and nonlinear effects of the light wave can be manipulated by engineering CD, in order to manipulate the temporal shape and frequency spectrum. Therefore, agile shapes of dispersion profiles, including typically wideband flat dispersion, are highly desired among various applications. In this study, we demonstrate a novel method for agile dispersion engineering of integrated photonic waveguide. Based on a horizontal double-slot structure, we obtained agile dispersion shapes, including broadband low dispersion, constant dispersion and slope-maintained linear dispersion. The proposed inverse design method is objectively-motivated and automation-supported. Dispersion in the range of 0–1.5 ps/(nm·km) for 861-nm bandwidth has been achieved, which shows superior performance for broadband low dispersion. Numerical simulation of the Kerr frequency comb was carried out utilizing the obtained dispersion shapes and a comb spectrum for 1068-nm bandwidth with a 20-dB power variation was generated. Significant potential for integrated photonic design automation can be expected.

## 1. Introduction

Photonic Integrated Circuit (PIC) is essential for integrated optical systems, and thus plays a key role in broad applications. In a PIC, the control of waveguide’s chromatic dispersion is of central importance in most of applications, which significantly influences the propagation of light field and the generation of various nonlinearity effects [1]. Waveguides with agile shapes of dispersion profiles could potentially constitute various functional devices. The targets of dispersion engineering may include fiber-induced dispersion compensation, tunable dispersion for mode locking of lasers, as well as many other relevant devices [2,3,4,5]. For instance, the generation of a light soliton comb requires a flat and low anomalous dispersion [6,7,8,9], while with normal dispersion, dark soliton, can be generated for obtaining improved optical combs [10,11,12,13]. Meanwhile, constant or extremely large dispersion may also be useful in applications of optical phase array, on-chip FWM/OPA process, and optical delay line [14,15,16,17,18]. In this way, agile dispersion with various shapes are highly desired for various on-chip applications.

The dispersion applied in a photonic integrated circuit is mainly designed by changing the materials and structures of a waveguide. One of the common ways to shape the dispersion is to change the dimensions of its structures [19,20], and find the target structure. This is a process of forward design, which may be quite difficult to execute due to the large amount of multi-parameter sweeping of simulation and optimization for complex structures. More complex structures of waveguides usually support the potential for better performance of dispersion design. The increased parameter inevitably leads to higher calculation difficulties. Therefore, a key point of dispersion engineering for a waveguide is to find an appropriate structure accurately and efficiently that possesses the target dispersion profile. By considering this situation, machine learning might be a valid method to achieve this objective. Through proper training of the Neural Network, mapping between input and output coefficients can be solved. Then, multiple parameters can be optimized simultaneously. This kind of inverse design method has been applied to quite a lot of photonic structure and fiber designs [21,22,23,24], and was found to be competent for supporting photonic design automation (PDA).

This paper proposes a novel inverse design approach to solve agile dispersion engineering by using Neural Network. A horizontal double slot waveguide structure is adopted with As2S3 and Si3N4 utilized. After generation of a certain amount of data set, the inverse design method cost little time for completing optimization. This process provides better accuracy, efficiency, and lower-complexity for fast and agile dispersion design of waveguides with reusability for design automation. Moreover, it can be extended to other kinds of optical devices. Therefore, great potential for design automation of PIC can be expected.

## 2. Principle of Dispersion Inverse Design

### 2.1. Waveguide Structure for Inverse Design

**Figure 1:**
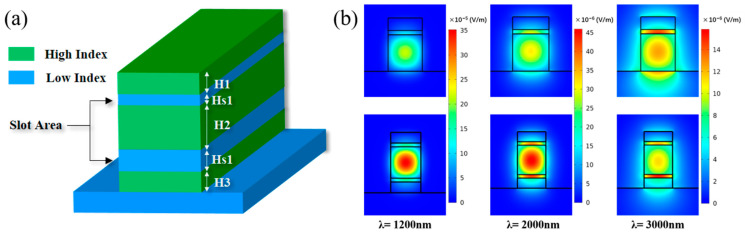

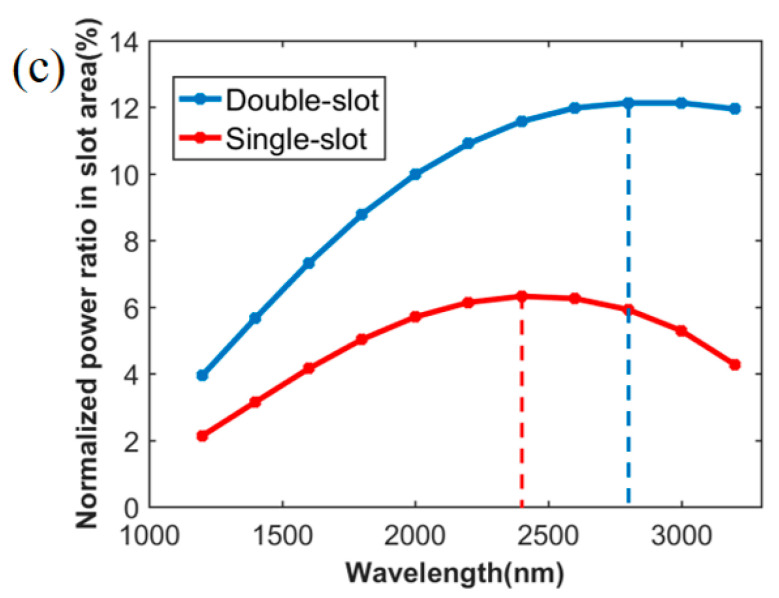
(**a**) Structures of horizontal double slot waveguide; (**b**) comparison of light field between single- and double-slot; (**c**): Comparison of the ratio for normalized power in slot area.

For the inverse design in this work, a horizontal double slot waveguide structure was adopted. Figure 1a shows six structural parameters of H1, H2, H3, Hs1, Hs2, and W, which have low refractive index materials lying between high index materials and the area of low index materials is called slot. In this work, the materials, As_2_S_3_ and Si_3_N_4_, are employed as different high index materials, respectively, and SiO_2_ is a low index material. The cladding is air. The material, As_2_S_3_, also named chalcogenide glass, has broadband infrared transparency and low linear and nonlinear propagation loss at C band and 2 μm band, and has been gradually applied for photonic integrated chips [25]. The material, Si_3_N_4_, is renowned for its extremely low propagation loss and relatively mature chip processing. Due to low TPA at C band, Si_3_N_4_ has been utilized among various nonlinear mechanisms. Two kinds of materials have broad applications and play important roles in photonic integration. In this way, universality can be verified by applying these materials to inverse design processes.

Moreover, slot structures have large degrees of freedom with multiple parameters for better dispersion engineering. They are able to confine intense light field in the slot area, which favors the generation of various nonlinear effects [26]. Furthermore, horizontal slots have greater fabrication tolerance due to smaller sidewall angle and less loss, compared with vertical slot [27]. In terms of double and single slot structures, the former could confine significant light field in a wider wavelength range, as shown in Figure 1b,c, which means greater potential to achieve agile dispersion in a broader waveband [28]. The wavelengths corresponding to maximum ratio of double- and single-slot are marked in the Figure by dot lines in Figure 1c.

Considering all the reasons mentioned above, we chose this horizontal double slot waveguide with materials of As_2_S_3_ and Si_3_N_4_ as target objective of inverse design.

### 2.2. Data Generation and Inverse Design Process

**Figure 2:**
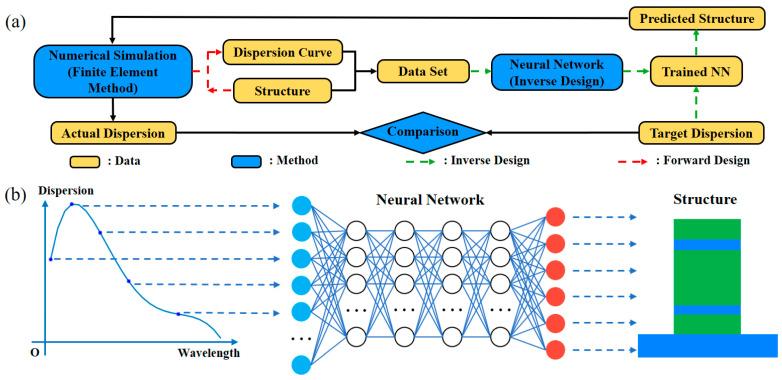
(**a**) Flow chart of the whole proposed inverse design method; (**b**) schematic principle of inverse design via Neural Network.

The entire design process was aided by Neural Network for dispersion engineering, as shown in Figure 2. The main data required involved the dispersion of a large amount of waveguide structures, which was varied with wavelength, forming a dispersion profile. The dispersion profiles with respect to wavelength λ are calculated by the second derivative of the effective index $n_{eff}$, which can be obtained by Finite Element Method (FEM) simulation using commercial software (COMSOL for example). The relationship between $n_{eff}$ and D can be described as follows in Equation (1):

(1) $$D=-\left(\lambda /c\right)\cdot \left(\partial^{2}n_{eff}/\partial \lambda^{2}\right).$$

Among the simulation experiments, material dispersion has been taken into account by scientists [29], as illustrated by Sellmeier Equation with different values for As_2_S_3_ and Si_3_N_4_.

The above equation clarifies the method for obtaining dispersion profiles, and one important part of the Neural Network-based inverse design is to obtain a proper data set, which contains different structural parameters of waveguides with corresponding dispersion profiles. The amount and variation range of a data set should be appropriately decided, otherwise it might waste time in generating data and the training network, or make the network fail to locate the intrinsic connection between the waveguide and its dispersion. Based on the data set, the network could be fed and trained.

In detail, given that it is almost impossible to obtain the analytical solution of the dispersion profiles, those in the data set were all numerical solutions. Each of the profiles contained many points, which are dispersion values corresponding to their wavelengths, and these points were calculated using Equation (1) mentioned above. The entire profile represents the dispersion variation of one set of structural parameters (six specific structural parameters form one set). For the essential data set, we use the six structural parameters [H1, H2, H3, Hs1, Hs2, W] and materials in simulation to obtain the dispersion values through indirect calculation from $n_{eff}$. Notably, we let H1 equal to H3, in order to reduce the computation in the research, as the change of H1 and H3 has a similar influence on waveguide’s dispersion [6]. Therefore, the data contained dispersion values varying with wavelength, λ, which represent the dispersion profile mentioned above, as well as the corresponding set of structural parameters. The entire data set consisted of thousands of pieces of data.

The data set is divided into input and output parts. The input part contains the discretely dispersion profile [D1, D2, …, Dn], while the output part contained the structural parameters [H1, H2, H3, Hs1, Hs2, W]. The variation range of those parameters in the data set is shown in Table 1. Further, discrete dispersion values from 1500 nm to 3500 nm for As_2_S_3_ waveguide and 1000 nm to 3000 nm for Si3N4 waveguide with an interval of 50 nm were calculated. Specific wavelength bands were selected and some of the values taken out to consider the target in the training process. Prior to training, proper preprocessing and normalization for the original data is essential. Here, we simulated the structures again where the data are identified as null values due to the calculation error of software. Then, we converted all the structural parameters into range [0, 1] via linear mapping (for example, for parameter W, the maximum value in W is converted to 1 and the minimum one is converted to 0), in order to increase the calculating efficiency. The same conversion was also applied to the dispersion values where the interval was [−1, 1]. After that, the input and output data were fed into the network, which is trained by adjusting the weight of each layer and comparing the error value continuously. The error value, here, means the difference between the predicted values from network and the actual values in the data set. The training process stops after specific number of epochs in the network. One epoch means a circulation in network training, after which the error value will gradually decreases. The number of epochs selected in our model is 200, considering both the efficiency and accuracy. There will be a convergence of error value if proper settings of network are made. After completing a careful search and attempts were made to identify the settings corresponding to a much lower convergence (stated in Section 2.3), an inverse design model was used to obtain proper mapping between discrete dispersion and structural parameters.

### 2.3. Network for the Inverse Design

After data generation, the core of inverse design followed—the construction and training of the network. Establishing a network with an appropriate structure is essential, as it is determinant of prediction accuracy. In our work, Deep Neural Network (DNN) was utilized. Both input and output data can be regarded as one-dimensional column vectors, feeding into the DNN. The structure of DNN is shown in Figure 2b. An open-source artificial Neural Network library Keras, based on Python, is applied. In terms of the network, there are four hidden layers and 200 neurons in each layer. Linear rectification function Relu and Sigmoid are applied in four hidden layers and an output layer, respectively. We selected the mean square error (MSE) as the loss function and Adam algorithm as the optimizer. The loss function, here, demonstrates how we measure the error value between predicted value and actual value, and the optimizer is the method by which the network decreases the error level. We also use the dropout layer to weaken over fitting [30,31,32]. The detailed settings of network are shown in Table 2.

In order to confirm the performance and accuracy of the trained network, we compared and identified the error value between the predicted parameters obtained by network and the actual value. Utilizing the test data set, an objective dispersion value set [D1, D2, …, Dn] could be formulated and placed into the trained network, then structural parameters could be predicted [H1, H2, H3, Hs1, Hs2, W], the corresponding dispersion value could be calculated. The actual structures of that dispersion are already known from the test data. Understanding whether the error between the predicted and actual structures are low or not is standard for confirming the performance of the trained network. Given that our aim was to obtain waveguide structure where the dispersion characteristic satisfied the specific profiles, this straightforward factor represented the error of structural parameters between predicted and actual values. Considering this, a comparison of predicted and actual values is displayed in the following part of Section 3.1 and Section 3.2.

For the inverse design process, a set of dispersion values (optimization aims) were used to meet the target performance, they were then fed into the trained network, in order to predict the corresponding structural parameters. Similarly, these predicted parameters were simulated to identify the real dispersion values, evaluate accuracy and the performance of the trained network. The procedure of inverse design is shown in the schematic of Figure 2b.

## 3. Results and Analysis

### 3.1. Inverse Design of As_2_S_3_ Waveguide

We generated the As_2_S_3_ waveguide data set with a scale of about 6000, and these data are divided into the train set and test set at a ratio of 80:20. Both the train and test set contain a number of complete pieces of data, which are used to train the network and check the performance of the network, respectively. Among the settings for the network, the ratio of 80:20 was one available option, and the algorithm automatically completes the division. The train set was used for training, including calculating the loss function and the optimizer to adjust the weight throughout the training process. Whereas, the test set was used for the network to check the values of loss function—it was not involved in the training process as it could only be used for testing. In this way, we ensured the reliability performance of the network evaluation via the test set. The results are displayed below.

For the inverse design of the As_2_S_3_ waveguide, we selected the target wavelength bands, ranging from 2000 nm to 2800 nm and from 1800 nm to 2900 nm, respectively (a wide and a narrow band), which means the input of network is on 9 [D1, D2, D3…, D9] dimensions or 11 [D1, D2, …, D11] dimensions, and the output was made up of 6-dimensional structural parameters [H1, H2, H3, Hs1, Hs2, W].

For the different target wavelength bands, where the structures of the network are the same, the different combinations of dispersion values were extracted from the data set to feed the network as input data. This also represents the efficiency and reusability of inverse design method. We did not use the entire dispersion values (1500 nm to 3500 nm for As_2_S_3_ waveguide and 1000 nm to 3000 nm for Si_3_N_4_ waveguide) due to the influence of the intrinsic zero dispersion of materials, which result in a rapid variation of waveguide’s dispersion inevitably. The reason for generating a data set with extra dispersion values was to retain the possibility of adjusting the target wavelength band freely by selecting different dispersion values as input of the network. This should not be time-consuming, and with different input data, the training process only lasts several tens to hundreds of seconds.

**Figure 3:**
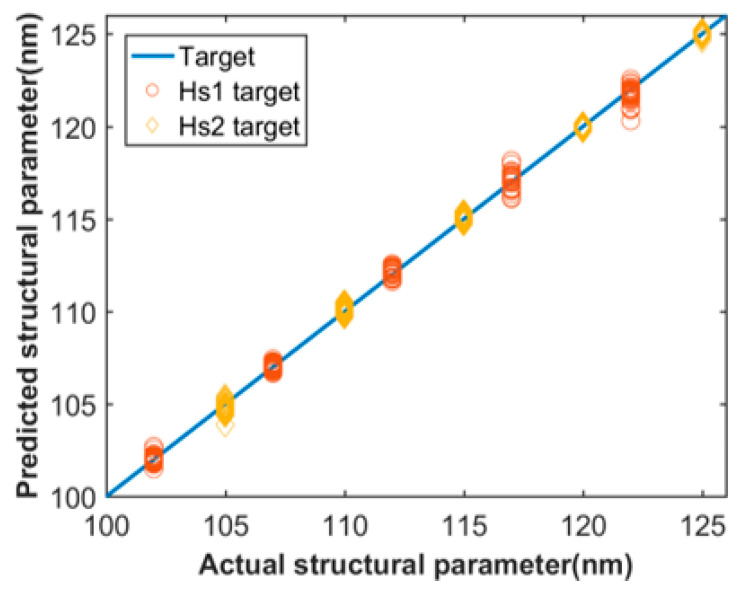
Actual and predicted data for As2S3 waveguide.

In Figure 3, the prediction of two of the six structural parameters were used to display the training results for examples. The target wavelengths, in this study, are 2000 nm to 2800 nm. The X-axis is the actual parameter, and the Y-axis is the predicted values obtained by setting target dispersion as the corresponding dispersion values from test set. This scatter diagram does not display all the testing results, which are in a large amount. As shown in Figure 3, most of points are close to the straight line of y = x with a small variation, which represents good performance of the network with the predicted values well close to actual values. We calculated the mean absolute error values of each structural parameters predicted by the trained network to check performance of the network more straightforward, as shown in Table 3. We found that the MAE values of those structural parameters are all less than 1 nm, which is highly within the tolerance for practical fabrication.

In order to achieve the practical application of photonic integrated circuit design, we set three goals for dispersion engineering: Broadband low dispersion in different wavelength bands, broadband constant dispersion with positive or negative values, and slope-maintained linear dispersion, where the target dispersion values are all 0, a certain constant value, and a linear function respectively. By applying the trained network, predicted structures corresponding to the target dispersion can be obtained. We place these structural parameters into simulation, calculate the actual dispersion profiles, then compare the actual and target dispersion for verification.

**Figure 4:**
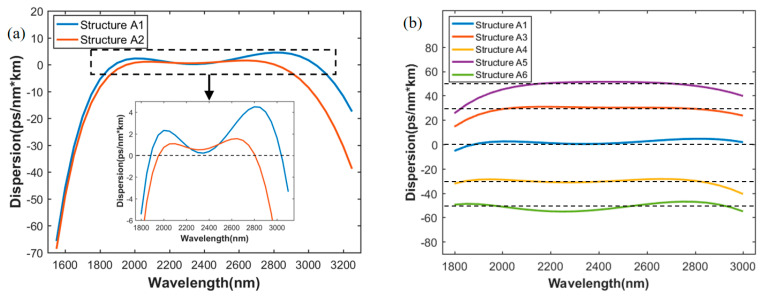

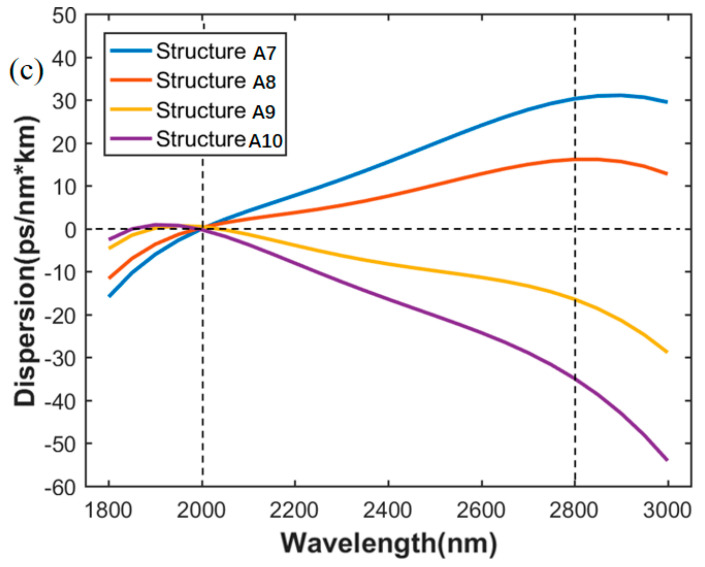
Inversely designed dispersion curves of As2S3 waveguides. (**a**) Broadband low dispersion for different target wavebands; (**b**) broadband maintained constant dispersion; (**c**) slope maintained linear dispersion.

The results of the inversely designed dispersion profiles of As2S3 waveguide obtained from trained Neural Network are shown in Figure 4. The detailed structural parameters, mentioned in Figure 4, is shown in Table 4.

In Figure 4a, the target wavebands are 1800–2900 nm and 2000–2800 nm respectively, which represents a wider band and a narrower band. Horizontal dotted line in the expanded Figure refers to the target dispersion values, which remained at zero, and represents the broadband low dispersion. The corresponding structures are A1 and A2, predicted by the network. Dispersion of A1 varies between 0–4.5 ps/(nm·km) for a 1154-nm bandwidth from 1885 nm to 3039 nm and dispersion of A2 varies between 0–1.5 ps/(nm·km) for an 861-nm bandwidth from 1945 nm to 2806 nm. The results of the predicted structures do not exactly follow the target bands due to the error of network. We found that, given that Structure A1 has a longer target band, its flatness of dispersion curve is not better than that of A2. However, it has a wider wavelength range with a relatively low dispersion. Compared with Reference [6], a much lower dispersion in a slightly narrower bandwidth was obtained by the proposed network.

Figure 4b,c show the results of broadband constant dispersion and slope maintained linear dispersion. The target waveband is 2000–2800 nm. Figure 4b shows five dispersion curves for structure A1, A3, A4, A5 and A6. The dispersion curve for A1 is for comparison. Broadband constant dispersion of 30, −30, 50, and −50 ps/(nm·km) can be predicted by the network. All five dispersion curves have wavebands of constant dispersion containing the target band, and the range of constant dispersion slightly exceeds the target band. As in Figure 4a, the horizontal dotted lines in Figure 4b refer to target values being fed into the network, corresponding to constant values mentioned above. Moreover, Figure 4c shows the linear dispersion curves with four different slopes, corresponding to structures A7, A8, A9, and A10 predicted by the network. The vertical dotted lines, lying on 2000 nm and 2800 nm, indicate the linear area, whereby the four dispersion slopes are maintained to be 0.04, 0.02, −0.02, −0.04 ps/(nm^2^·km), respectively. This kind of linear dispersion may be useful for compensation in fiber systems.

### 3.2. Inverse Design of Si_3_N_4_ Waveguide

Our proposed dispersion engineering method can also be applied to Si3N4 double slot waveguide, and it proves universality in photonic waveguide design. The process of network training is basically similar to that of As_2_S_3_ waveguide, but the new data set needs to be generated for theSi_3_N_4_ waveguide. The selected target wavelength band range from 1300 nm to 2200 nm and 1200 nm to 2400 nm, a wider band and a narrower band. Different material dispersion and modal refractive index should be expected for Si_3_N_4_ waveguide.

**Figure 5:**
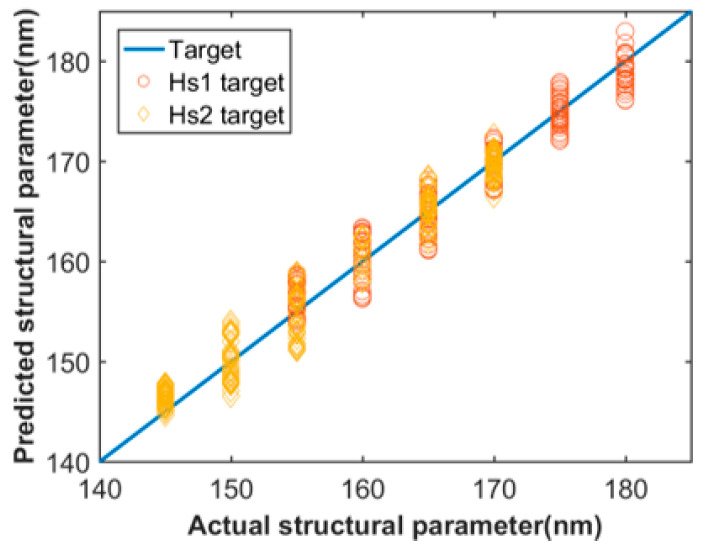
Actual and predicted data for Si3N4 waveguide.

**Figure 6:**
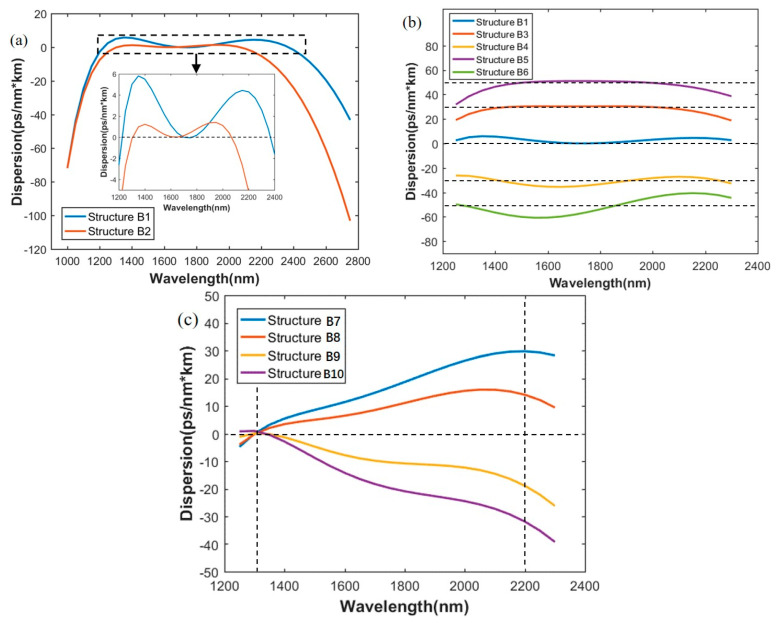
Inversely designed dispersion curves of Si3N4 waveguides. (**a**) Broadband low dispersion for different target wavebands; (**b**) broadband maintained constant dispersion; (**c**) slope maintained linear dispersion.

The scatter diagram containing the prediction of two parameters for Si_3_N_4_ waveguide is shown in Figure 5, and the mean absolute error values of the results for Si_3_N_4_ waveguide are shown in Table 5. The target band is 1300 nm to 2200 nm. The MAE values are also at a lower level, indicating good performance of prediction. However, the performance of the inverse design for Si3N4 waveguide is not as good as that of As_2_S_3_ waveguide, in terms of the predict accuracy in scatter diagram and mean absolute error value in Table 5. In fact, there might be a best interval for scale of data set and variation range of the parameters in the data set, if deviated from this interval, the performance of network will decrease when other conditions remain unchanged. We attempted to find the best interval for Si_3_N_4_ waveguide, but a difference exists, maybe due to the sub-par interval for that of Si_3_N_4_ waveguide. Nonetheless, the results in Figure 6 show that the network for Si_3_N_4_ waveguide also possess satisfying performance, thus the difference between Table 3 and Table 5 can be tolerated. The detailed structural parameters mentioned in Figure 6 are shown in Table 6.

As for the predicting results, in Figure 6a, for structure B1, the broadband low dispersion varies from 0 to 5.8 ps/(nm·km) ranging from 1226 nm to 2368 nm for a 1142-nm bandwidth and for structure B2, dispersion varies from 0 to 1.4 ps/(nm·km) from 1306 nm to 2067 nm for a 761-nm bandwidth. Horizontal dot lines in zoom-in figure are the target dispersion values, all zero here, which represents broadband low dispersion. These well performed results in terms of much broader and lower dispersion compared with Reference [7] would be highly useful for broadband nonlinear processing applications on Si3N4 platform.

Similar to As_2_S_3_, dispersion engineering, with maintained constant values or maintained dispersion slopes, is also carried out using the proposed method, as shown in Figure 6b,c. Constant dispersions of 30, −30, 50, and −50 ps/(nm·km), ranging from 1300 nm to 2200 nm with small variations, are obtained by structure B3 to B6. While, the horizontal dot lines are target values, corresponding to the certain dispersion. The dispersion slopes of 0.04, 0.02, −0.02, −0.04 ps/(nm^2^·km) from 1300 nm to 2200 nm are maintained, as indicated by the vertical dot lines, and obtained by Structure B7 to B10, as shown in Figure 6c.

### 3.3. Influence of Sidewall Angle in Fabrication

**Figure 7:**
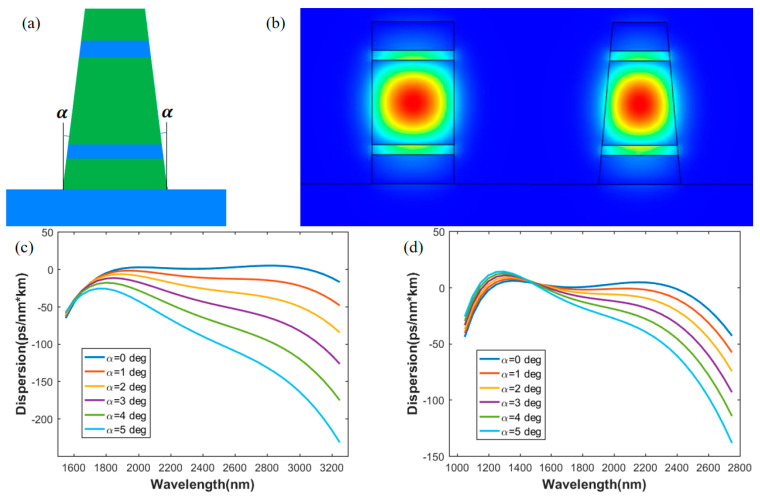
(**a**): Waveguide with sidewall angles; (**b**) fundamental modes for waveguides with sidewall angle of 0 and 5 degrees; (**c**) dispersion curves with different sidewall angles for Structure A1; (**d**) dispersion curves with different sidewall angles for Structure B1.

Considering the fabrication of the horizontal double slot waveguide, there always exists a sidewall angle α, which means the wall of the waveguide is not perfectly vertical, as shown in the Figure 7a. This may influence the light field distribution and then correspondingly change the waveguide dispersion. As shown in Figure 7b, the waveguide in the left is perfectly vertical and the waveguide on the right has a sidewall angel of 5 degrees.

Using Structure A1 and B1 as examples, the dispersion of the waveguide, with different sidewall angles from 0 to 5 degrees, was investigated. The results are shown in Figure 7c,d, respectively. We found that when the sidewall angle increases, the dispersion curves of the waveguide bend slightly. The dispersion of As_2_S_3_ waveguide changes more significantly than that of Si_3_N_4_ waveguide. A relatively high precision for As_2_S_3_ and Si_3_N_4_ waveguide fabrication needs to be maintained to keep the sidewall angle at a small level, in order to obtain the designed dispersion profile.

The fabrication of horizontal slot waveguide has already been studied in several works [33,34]. In view of the silicon nitride horizontal slot waveguide, the thin film of silicon nitride and silica can be deposited on silica wafer in the specific order via low pressure or plasma enhanced chemical vapor deposition (LPCVD/PECVD). The depositing thickness of film can be controlled by the duration time of deposition. After that, a dry etching method, such as reactive ion etching (RIE), can be applied to complete the processing of waveguide, which will result in a high performance of fabrication.

### 3.4. Generation of Frequency Combs via Double Slot Micro-Ring Resonator

It can be inferred from the study in [29] that a well-designed flat and low anomalous dispersion is conducive for generating the Kerr frequency comb with board bandwidth and small power variation. We formulated micro-ring resonators with double-slot structure, with B1 and B2 specific structures. Then, we adopted their dispersion curve into a simulation of Kerr frequency comb, in order to verify the performance of practical application via inverse design process.

The dynamics of comb generation in micro-ring resonators is well-described by the Lugiato-Lefever equation, which can be described as Equation (2) below [35,36]:

$$\frac{\partial {\tilde{A}}_{\mu }\left(t\right)}{\partial t}=\left(-\frac{\kappa }{2}+i\left(2\pi \delta_{0}\right)+iD_{\mathrm{int}}\left(\mu \right)\right){\tilde{A}}_{\mu }$$

(2) $$-igℱ{\left[{\left|A\right|}^{2}A\right]}_{\mu }+\sqrt{\kappa_{ex}}\cdot s_{in}$$

where ${\tilde{A}}_{\mu }$ and A means spectral and temporal envelopes of light field in resonators, respectively, and their relationship follows the Fourier transform. While, κ is loss rate of the resonator and $\kappa_{ex}$ is coupling rate between bus waveguide and resonator, g is Kerr frequency shift, and ${\left|s_{in}\right|}^{2}$ represents the pump power. $D_{\mathrm{int}}\left(\mu \right)$ means the integrated dispersion of μth frequency component and this can be calculated through the dispersion curve obtained above. $\delta_{0}$ is pump detuning. In our simulation, FSR of comb is set to be 200 GHz, corresponding to L=850 μm and R=135 μm. The loss of waveguide (Si3N4) is set to be 0.1 dB/cm and the resonator is assumed critical coupling, which means $\kappa =\kappa_{ex}$. The Q value of resonator is $6.5\times 10^{6}$. The power and wavelength of pump is 1 W and 1800 nm. The pump detuning is 56 times of resonator FWHM, around 4.88 GHz.

**Figure 8:**
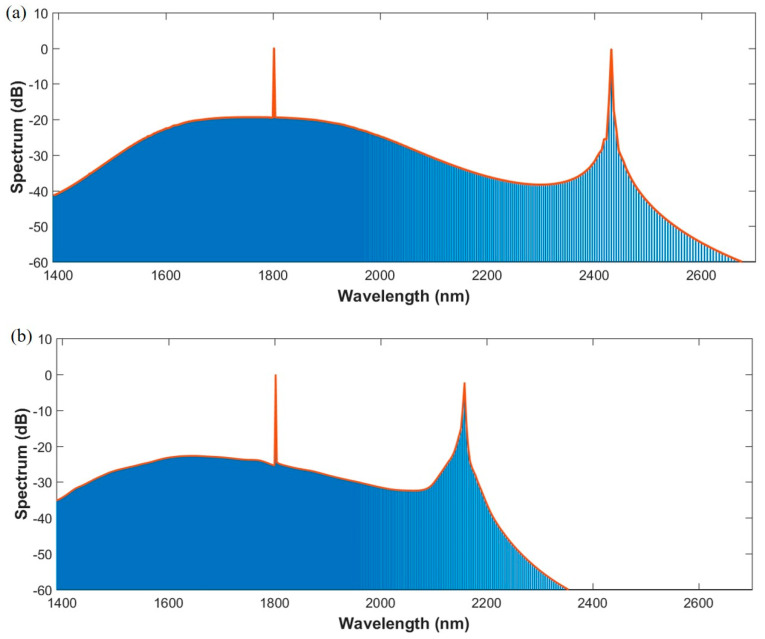
(**a**) Spectrum of Kerr frequency comb generated from micro-ring resonator with Structure B1; (**b**) spectrum of Kerr frequency comb generated from micro-ring resonator with Structure B2.

Based on the conditions mentioned above, the flat and broad spectrums of Kerr frequency comb are obtained from Structure B1 and B2, as shown in Figure 8, in order to compare influence of dispersion. The peak of spectrum in the longer wavelength area is generated because of the zero-value of integrated dispersion, according to Reference [9]. The spectrum in Figure 8a has a wide wavelength range and the power variation is relatively smooth. Quantificationally speaking, for a 10-dB power variation, the bandwidth of spectrum is measured to be 564 nm from 1515 nm to 2079 nm. For a 20-dB variation, the bandwidth of spectrum is measured to be 1068 nm from 1415 nm to 2483 nm. In Figure 8b, the spectrum of B2 possesses smaller power variation around the narrower band due to the smaller dispersion. For a 10-dB power variation, the band width of spectrum is measured to be 777 nm from 1416 nm to 2193 nm. It lacks bandwidth due to the narrower range of low dispersion. In addition, comb spectrum of strip waveguide is an envelope of square of hyperbolic secant [9]. In comparison, the spectrum of double slot waveguide has the potential to extend to low power variation. In practical terms, this could be adjusted according to the actual demands.

## 4. Conclusions

In this work, a Neural Network aided inverse design method is demonstrated for agile dispersion engineering of a horizontal double slot waveguide structure. The proposed objective-motivated, automation-supported inverse design method provides rapid optimization with high performance. Broadband low dispersion, constant dispersion and slope maintained linear dispersion were achieved for As_2_S_3_ and Si_3_N_4_ waveguides. These well-performed studies achieved results of versatile dispersion profiles that would be highly useful for broad applications. We believe this can be extended to very broad photonic devices to obtain ultimate performance far beyond dispersion. Significant potential can be expected for photonic design automation of integrated circuit.

## Figures and Tables

**Table 1:** Structure parameter ranges of As2S3 and Si3N4 waveguides.

Material	Parameter	Min (nm)	Max (nm)
As_2_S_3_	W	880	975
	Hs1	97	122
	Hs2	102	125
	H1	310	340
	H2	945	975
	H3	310	340
Si_3_N_4_	W	1120	1280
	Hs1	155	180
	Hs2	145	170
	H1	300	325
	H2	950	975
	H3	300	325

**Table 2:** Settings of the Neural Network.

Used NN Library	Hidden Layers	Neurons per Layer
Keras	4	200
**Activation function**	**Loss function**	**Optimizer**
Relu and Sigmoid	Mean Square Error	Adam algorithm

**Table 3:** Mean Absolute Error of predicted structural parameters.

Mean Absolute Error (MAE)	Hs1	Hs2	H1&H3	H2	W
As2S3 Slot Waveguide	0.24	0.16	0.38	0.45	0.55

**Table 4:** The structural parameters of mentioned dispersion engineering waveguide.

Structure (nm)		Hs1	Hs2	H1&H3	H2	W
As2S3 Slot Waveguide	A1	111.5	112	330	961	930
	A2	114.5	105	330	962	907.5
	A3	104	114	330	1010	911
	A4	101	116	329	919	880
	A5	98	114	323	1037	893
	A6	107	116	329	892	898
	A7	103.5	102	328	960	923.5
	A8	111	101.5	325	958	915
	A9	100	123	330	957	880
	A10	98	126	330	961	847

**Table 5:** Mean Absolute Error of predicted structural parameters.

Mean Absolute Error (MAE)	Hs1	Hs2	H1&H3	H2	W
Si3N4 Slot Waveguide	1.27	1.88	1.13	0.99	2.33

**Table 6:** The structural parameters of mentioned dispersion engineering waveguide.

Structure (nm)		Hs1	Hs2	H1&H3	H2	W
Si3N4 Slot Waveguide	B1	176.5	171.5	307	941	1309
	B2	161	149	318	939.5	1131
	B3	162	159.5	298	979	1216.5
	B4	161	172	327	889	1189
	B5	162	156	299	1035	1216.5
	B6	175	159	325	855	1189
	B7	165	147.5	307	953	1293
	B8	161	151	306.5	937	1218
	B9	168	156	324	938	1126
	B10	183	155	325	935	1120

## Data Availability

Not applicable.
